# Supplementary figures and images for: The impact of rearing environment on C. elegans: Phenotypic, transcriptomic and intergenerational responses to 3D enriched habitats
Source: bioRxiv. 2025 Sep 9:2025.09.07.674770. Preprint. [Version 1] doi: 10.1101/2025.09.07.674770 (PMC12439878; doi:10.1101/2025.09.07.674770)

**A**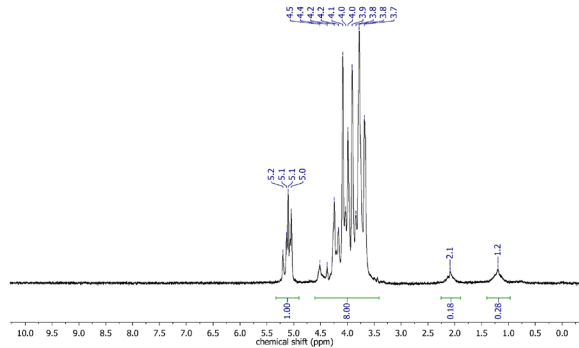**B**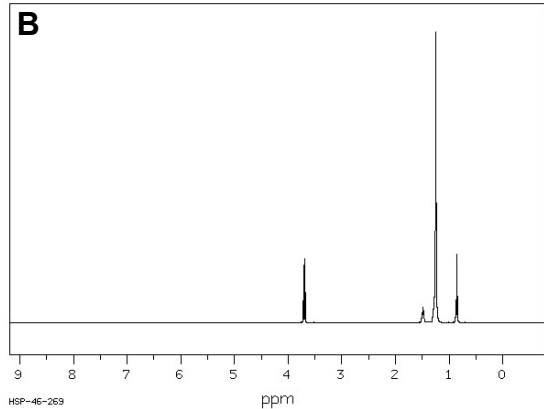

Supplement: Supplement 1 — Figure S1. 1H HR-MAS NMR analysis confirms the absence of detergent residues. (A) Full spectral region of the decellularized apple scaffold. (B) Full spectral region of the decellularizing agent sodium dodecyl sulfate (SDS) (National Institute of Advanced Industrial Science and Technology, 1999). [file media-1.pdf]

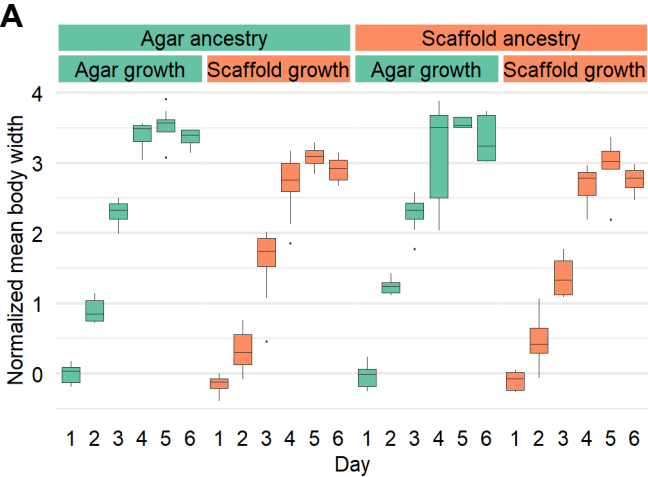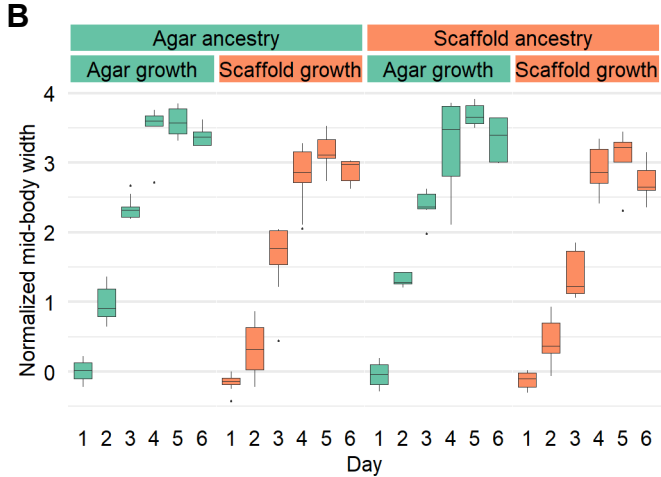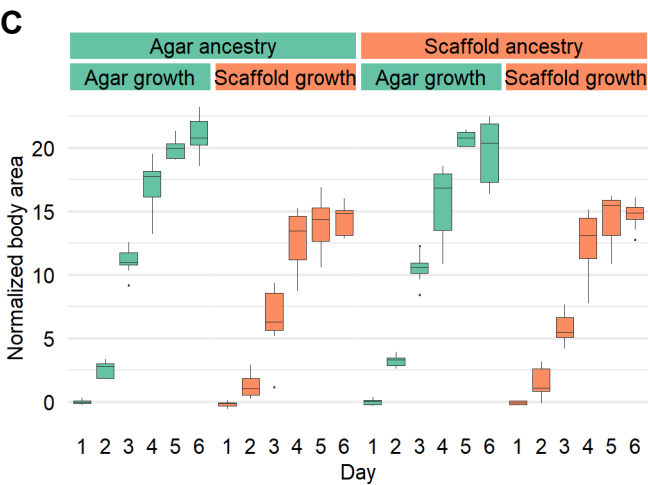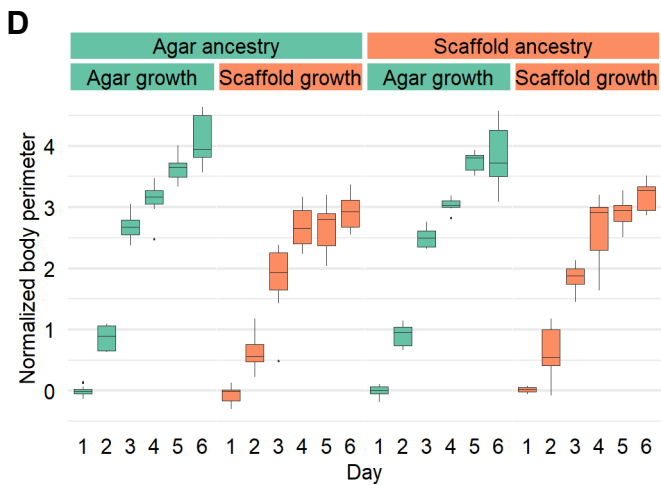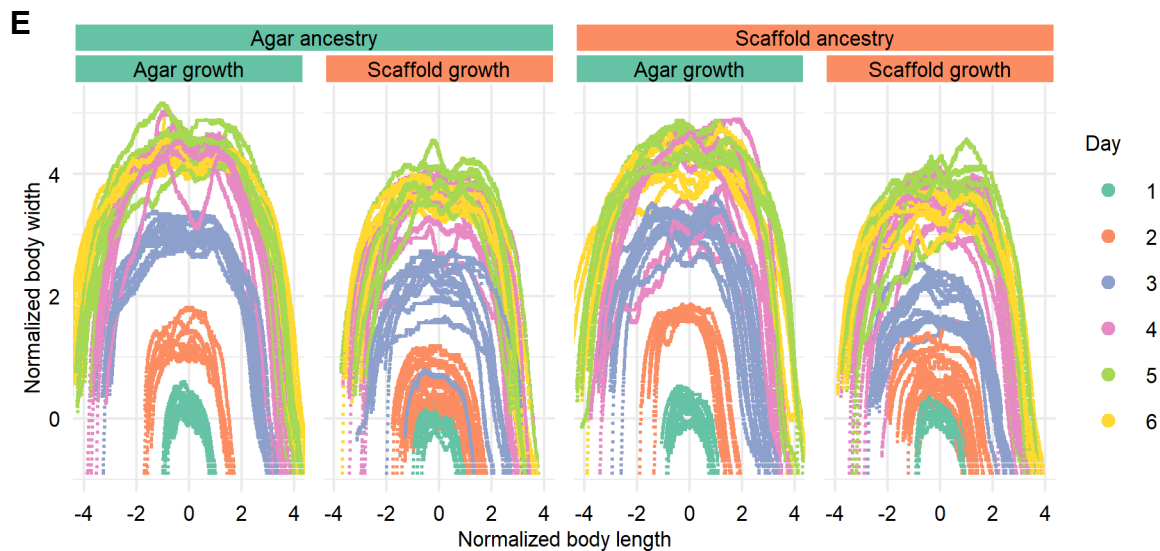

Supplement: Supplement 2 — Figure S2. Developmental dynamics of body morphology. (A) Mean body width, (B) mid-body width, (C) body area, and (D) body perimeter by day. (E) Body width along body length with mid-body centered at 0. Colors represent days post-hatching. All measured values are normalized to the mean of agar:agar worms on day 1. Sample sizes: same as Figs 2B–D. [file media-2.pdf]

**A**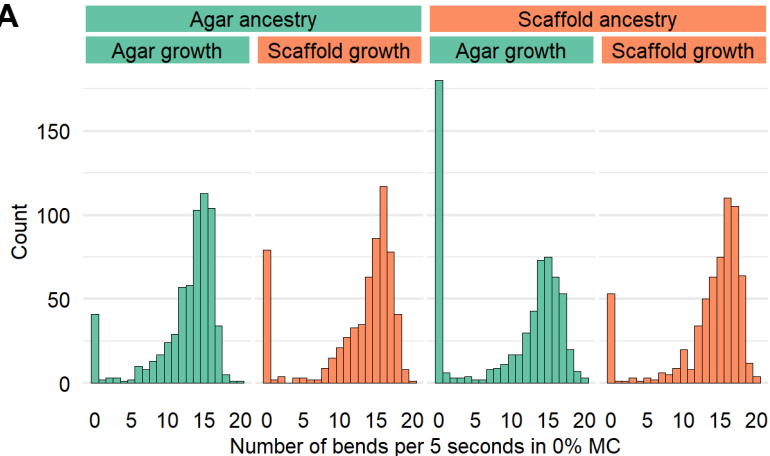**B**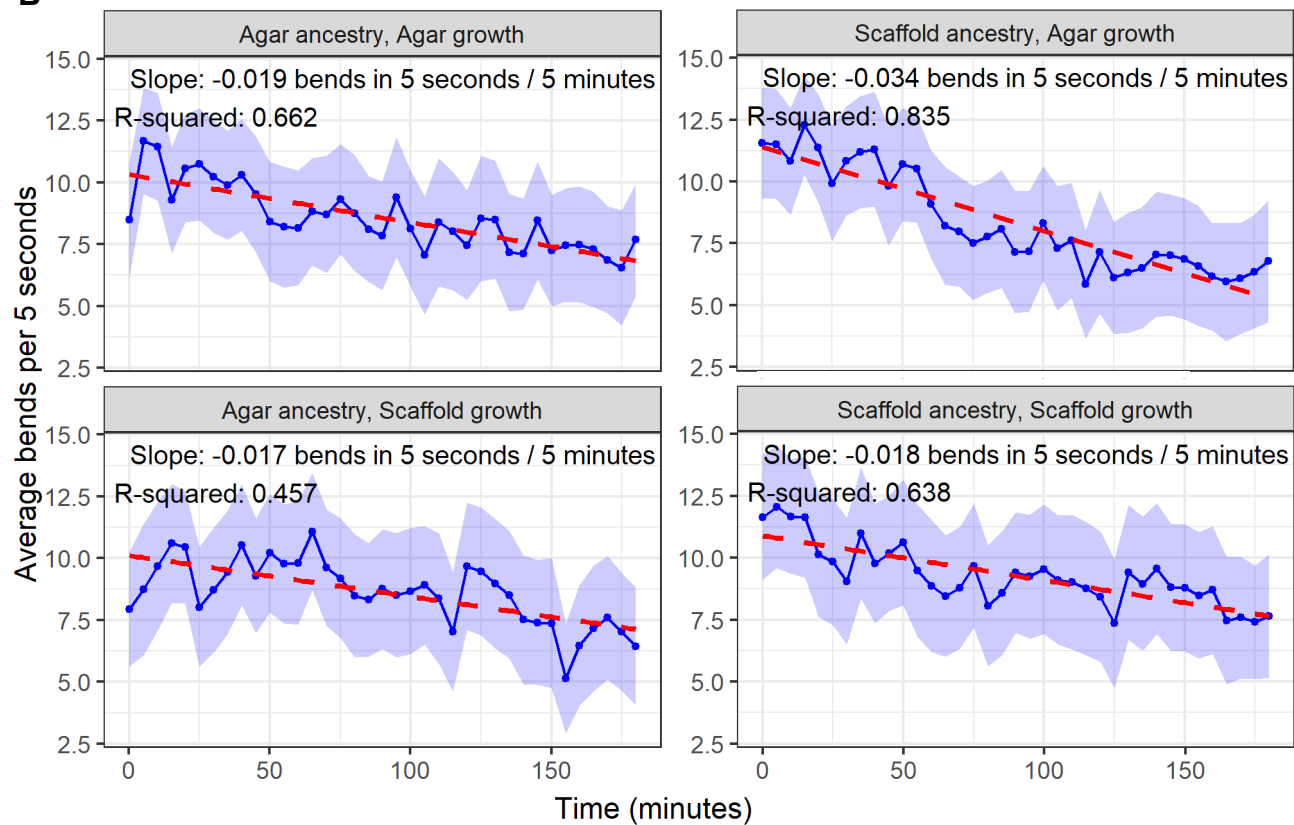

Supplement: Supplement 3 — Figure S3. Long-term swimming patterns reveal shifts in activity. (A) Histogram of number of swimming bends per 5 seconds in 0% MC. (B) Average number of bends per 5 seconds for each time point (point and line) with 95% confidence interval (ribbon) and linear regression (dashed line), in 0% MC. Text insets: slope and R-squared values. Sample sizes: same as Figs 3A–D. [file media-3.pdf]

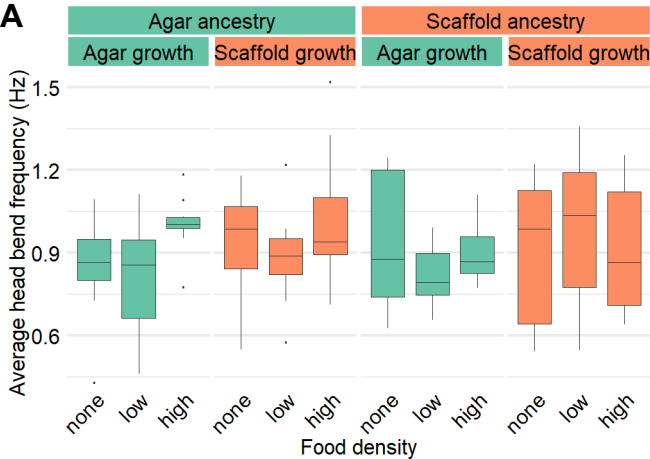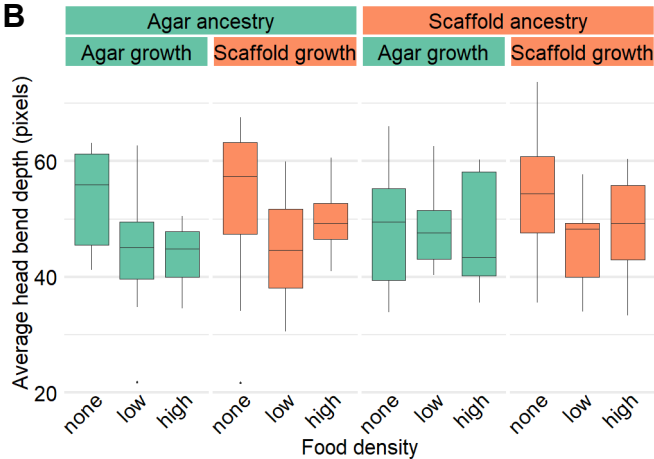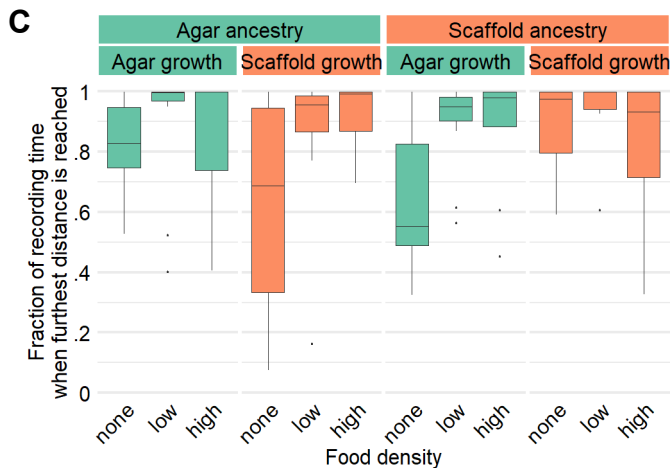

Supplement: Supplement 4 — Figure S4. Altered temporal exploration patterns during crawling. (A) Average head bend frequency (Hz) by food density. (B) Average head bend depth in pixels by food density. (C) Fraction of the recording period when the furthest Euclidean distance from the start point was reached by food density. Sample sizes: same as Fig. 6. [file media-4.pdf]

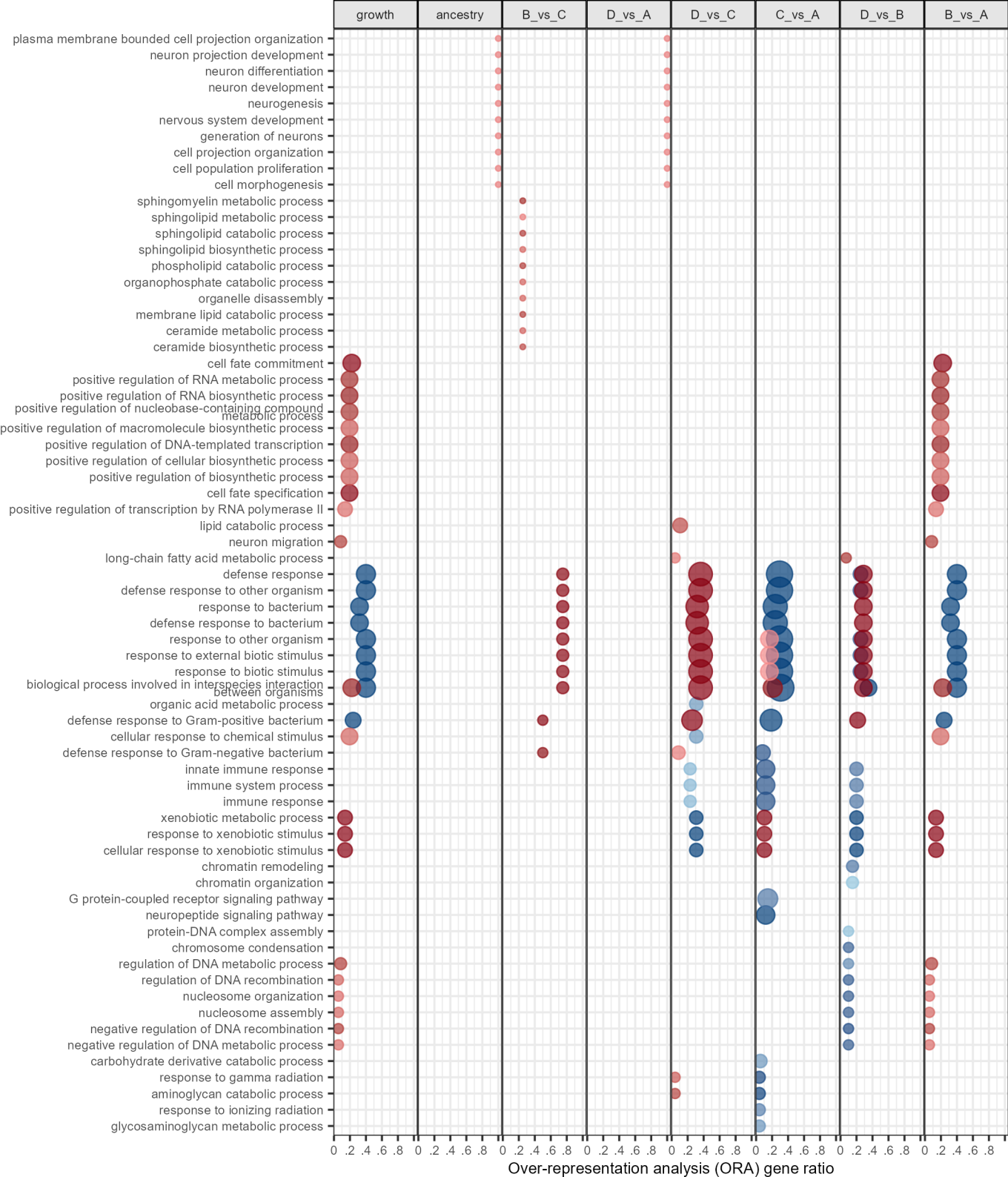

Supplement: Supplement 5 — Figure S5. Detailed over-representation analysis of Biological Process GO terms. Same legend as Fig. 8. Facet labels correspond to: “growth”: scaffold versus agar growth factor; “ancestry”: scaffold versus agar ancestry factor; “A”: agar:agar condition; “B”: agar:scaffold condition; “C”: scaffold:scaffold condition; “D”: scaffold:agar condition. [file media-5.pdf]

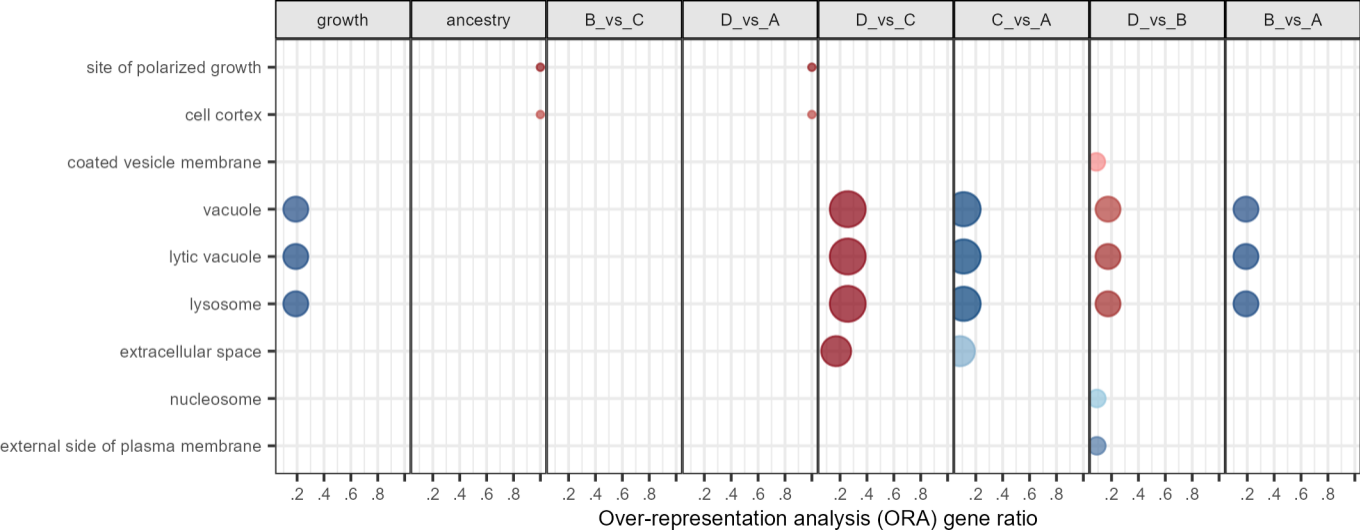

Supplement: Supplement 6 — Figures S6. Detailed over-representation analysis of Cellular Component GO terms. Same legend as Fig. 8. Same facet labels as Fig. S5. [file media-6.pdf]

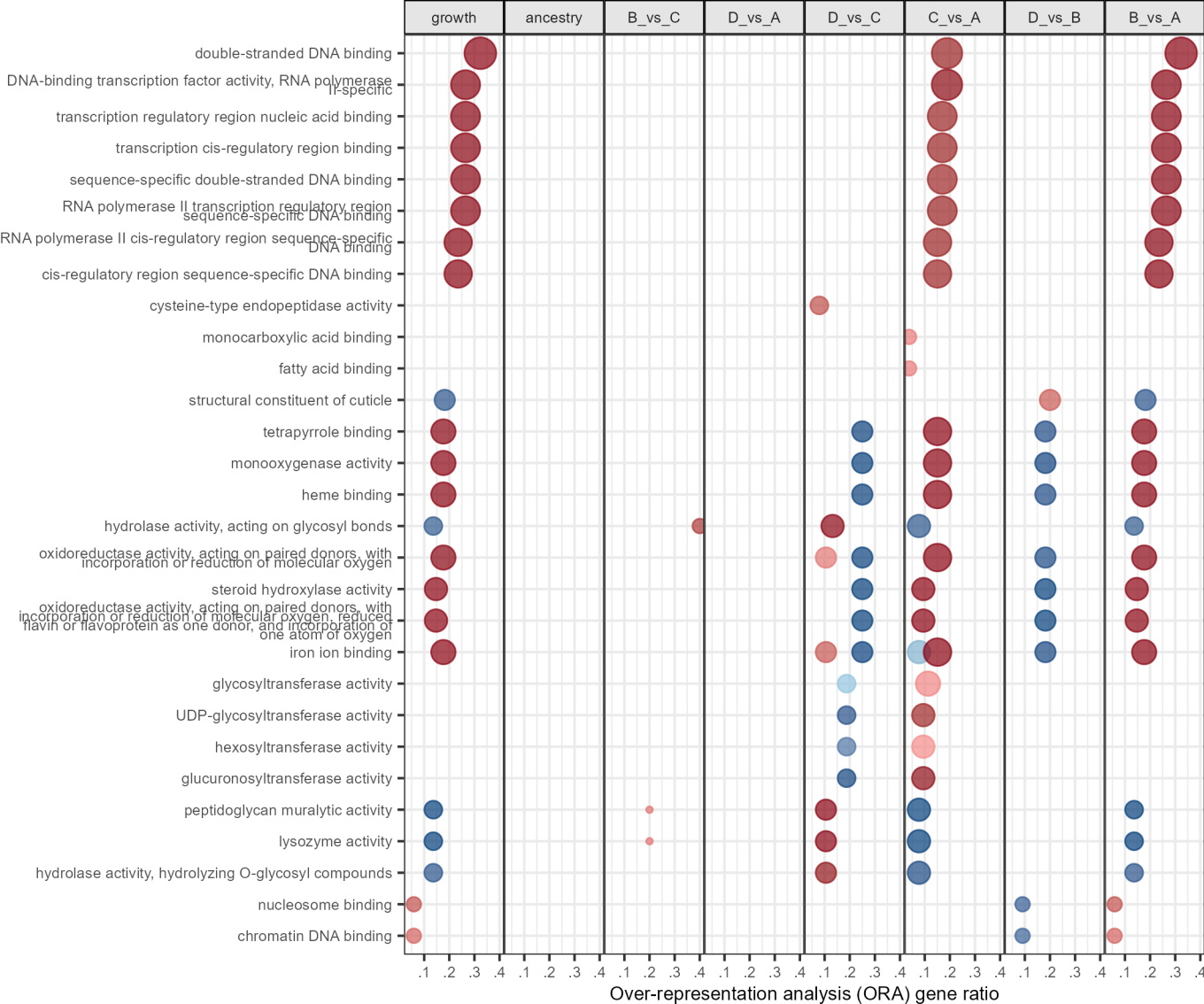

Supplement: Supplement 7 — Figures S7. Detailed over-representation analysis of Molecular Function GO terms. Same legend as Fig. 8. Same facet labels as Fig. S5. [file media-7.pdf]
