# Supplementary material for: The impact of rearing environment on C. elegans: Phenotypic, transcriptomic and intergenerational responses to 3D enriched habitats
Source: bioRxiv. 2025 Sep 9:2025.09.07.674770. Preprint. [Version 1] doi: 10.1101/2025.09.07.674770 (PMC12439878; doi:10.1101/2025.09.07.674770)
Supplement: Supplement 8 — Figure S8. Expression profile of genes commonly associated with calorie-restriction in C. elegans. Log2fold change value of 25 genes commonly associated with calorie-restriction (lgg-1, col-146, flp-21, acs-20, ragc-1, sbp-1, drr-2, T24B8.5, pha-4, aak-2, skn-1, eat-2, daf-2, clk-1, clk-2, clk-3, gro-1, zip-2, atf-7, nhr-62, ptr-8, nhr-23, sfa-1, sir-2.1, atx-2) ordered by descending p-adjusted value. Dashed lines represent p-adj < 0.05. Absence of dashed line means no gene reached the significance threshold. Same facet labels as Fig. S5. [file media-8.pdf]

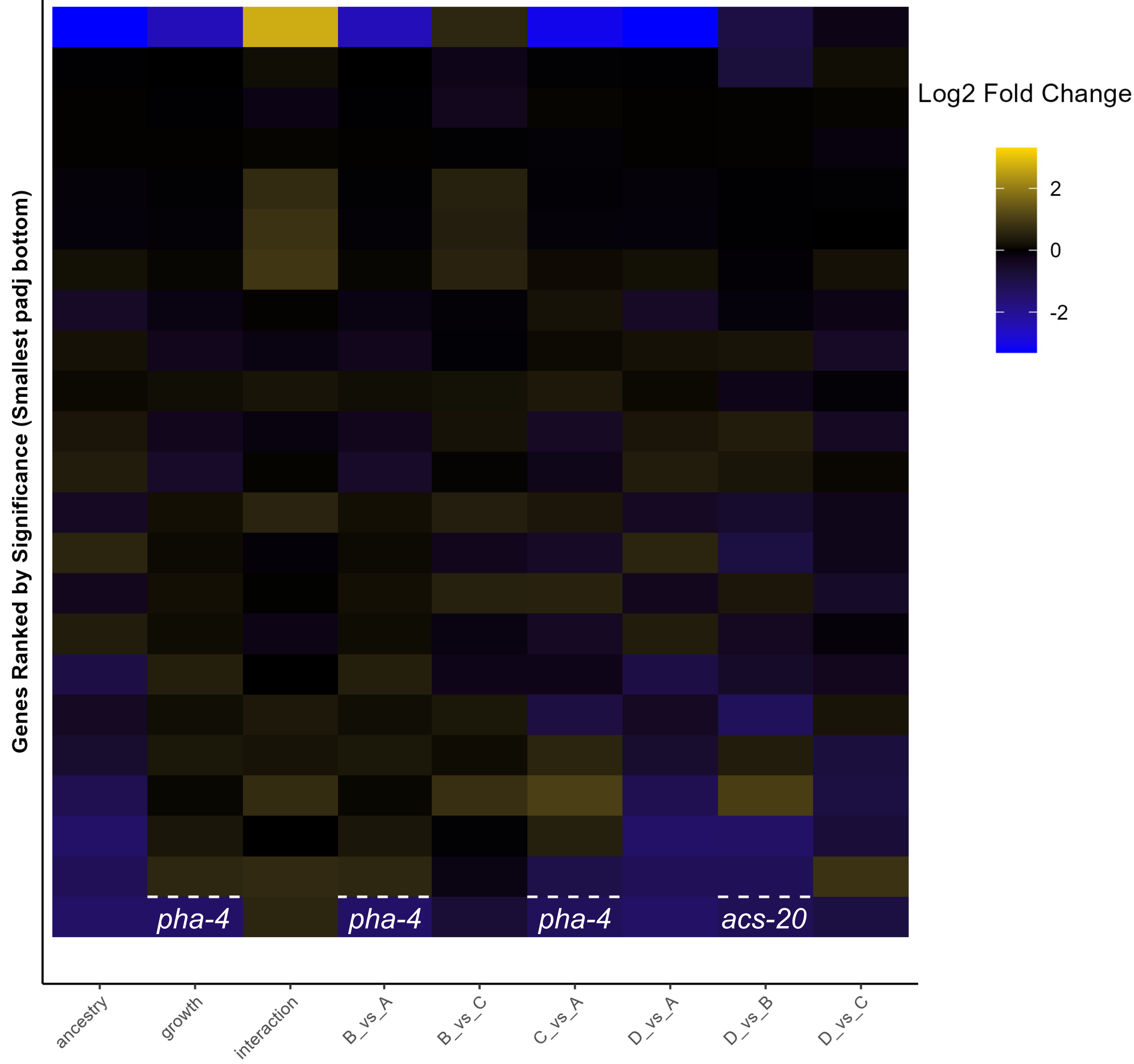

Log2 Fold Change
2
0
-2
Genes Ranked by Significance (Smallest padj bottom)
pha-4
pha-4
pha-4
acs-20
ancestry
growth
interaction
B_vs_A
B_vs_C
C_vs_A
D_vs_A
D_vs_B
D_vs_C
